# Steroid sulfatase deficiency causes cellular senescence and abnormal differentiation by inducing Yippee-like 3 expression in human keratinocytes

**DOI:** 10.1038/s41598-021-00051-w

**Published:** 2021-10-21

**Authors:** Hyoung-Seok Baek, Tae-Uk Kwon, Sangyun Shin, Yeo-Jung Kwon, Young-Jin Chun

**Affiliations:** College of Pharmacy and Center for Metareceptome Research, Chung-Ang University, Seoul, Republic of Korea 06974

**Keywords:** Biochemistry, Biomarkers

## Abstract

Human steroid sulfatase (STS) is an enzyme that catalyzes the hydrolysis of dehydroepiandrosterone sulfate (DHEAS), estrone sulfate (E1S), and cholesterol sulfate. Abnormal expression of STS causes several diseases including colorectal, breast, and prostate cancer and refractory skin disease. In particular, accumulation of intracellular cholesterol sulfate by STS deficiency leads to a skin disorder with abnormal keratinization called X-linked ichthyosis (XLI). To determine the detailed mechanisms of XLI, we performed RNA sequencing (RNA-seq) analysis using human keratinocyte HaCaT cells treated with cholesterol and cholesterol sulfate. Of the genes with expression changes greater than 1.5-fold, Yippee-like 3 (YPEL3), a factor expected to affect cell differentiation, was found. Induction of YPEL3 causes permanent growth arrest, cellular senescence, and inhibition of metastasis in normal and tumor cells. In this study, we demonstrate that YPEL3 expression was induced by STS deficiency and, using the CRISPR/Cas9 system, a partial knock-out (STS^+/−^) cell line was constructed to establish a disease model for XLI studies. Furthermore, we show that increased expression of YPEL3 in STS-deficient cell lines promoted cellular senescence and expression of keratinization-related proteins such as involucrin and loricrin. Our results suggest that upregulation of YPEL3 expression by STS deficiency may play a crucial role in inducing cellular senescence and abnormal differentiation in human keratinocytes.

## Introduction

Steroid sulfatase (STS) is responsible for catalytic reactions that convert sulfated steroids into active steroids such as dehydroepiandrosterone (DHEA), estrone (E1), and cholesterol^1–4^. It is known that abnormal expression or deficiency of STS causes various types of diseases including endometriosis, uterine fibrosis, cancer, and skin disease^4–8^. In particular, STS deficiency causes a refractory skin disease called XLI^9–11^. XLI is a disease which causes excessive keratinization, and occurs more often in males than in females because it results from a deletion or mutation of the STS gene in the X chromosome^12^. Russell et al. showed that accumulation of intracellular cholesterol sulfate interferes with the normal function of transglutaminase 1 (TGM1) and induces XLI^13^. However, the detailed mechanisms of XLI are not yet fully understood.

The excessive keratinization occurs as a result of abnormal differentiation in keratinocytes. In particular, the content of involucrin (INV) and loricrin (LOR) in cornified envelop is relatively high in differentiating keratinocytes^14,15^. Thus, elucidating new factors to regulate expression of INV and LOR that promote differentiation is important in finding a therapeutic approach to skin diseases such as XLI.

Previous studies have shown that YPEL3 induces cellular senescence, permanent growth arrest, or inhibition of metastasis in various cells^16–18^. A previous study has also shown that p53 induces expression of YPEL3, and suppression of YPEL3 expression in osteosarcoma cells is correlated with CpG hypermethylation on p53 binding sites of the YPEL3 promoter^16^. Tuttle et al.^19^ showed that removal of estrogen in MCF-7 cells increases expression of YPEL3 and causes cellular senescence, independently of the transcriptional activation by p53.

In this study, we investigated the role of YPEL3 in STS deficiency. We show that YPEL3 is upregulated by STS deficiency, and that it may play a crucial role in causing keratinization and senescence in this disease. Therefore, YPEL3 may be a therapeutic target for STS-dependent skin disorders occurring in human keratinocytes.

## Results

### Cholesterol sulfate induces YPEL3 expression in HaCaT cells

**Figure 1:**
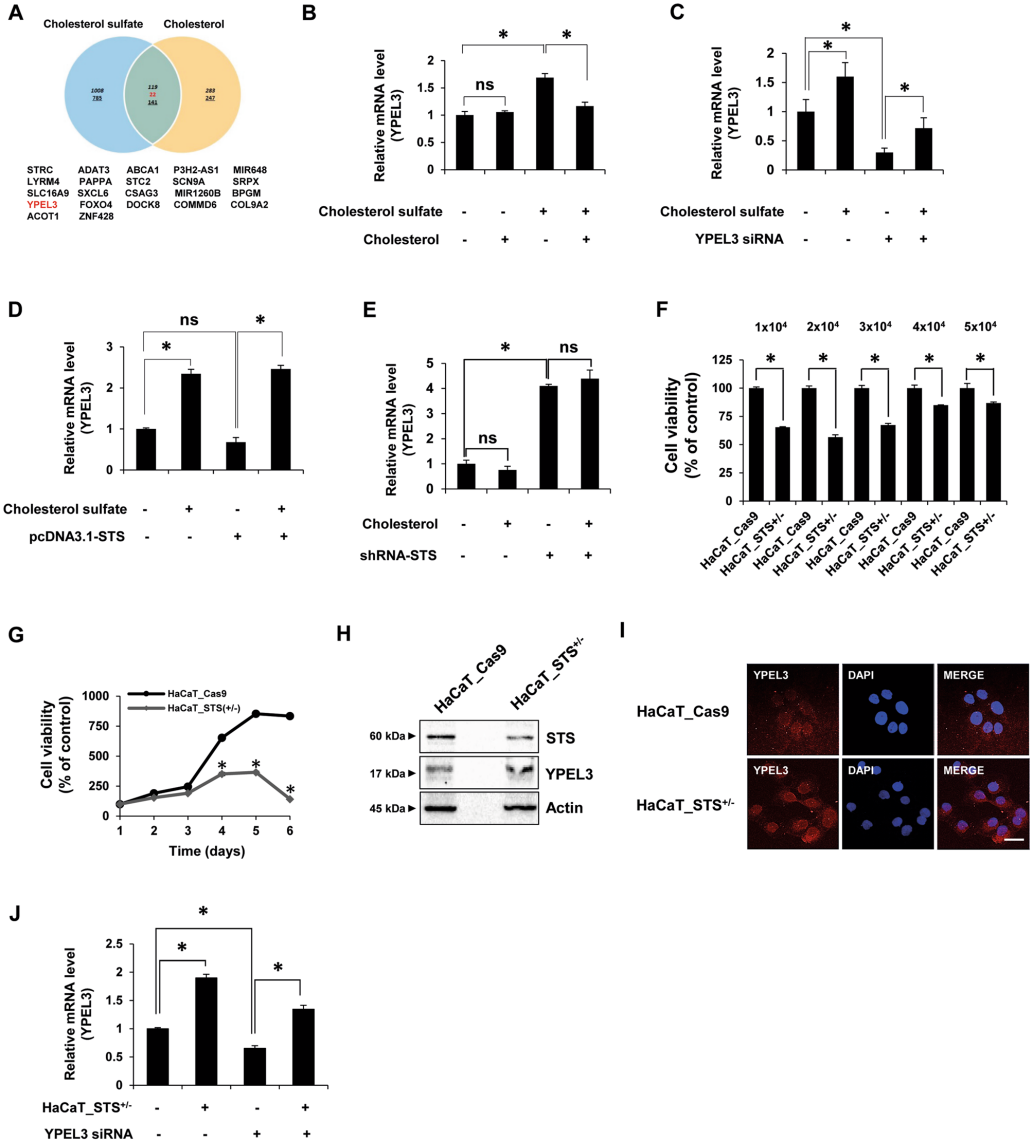
Cholesterol sulfate and STS deficiency induce YPEL3 expression in HaCaT cells. (**A**) Venn diagram of differentially expressed genes in the human keratinocytes HaCaT cell line after treated with cholesterol (20 μg/mL) or cholesterol sulfate (20 μg/mL) for 24 h. The 22 genes that are most significantly up-regulated and down-regulated in the RNA-seq data sets are listed beside the venn diagram. Cut off ≥ 1.5 fold. (**B**–**E**) Real-time qPCR was performed to detect the expression of YPEL3 mRNA. (**B**) HaCaT cells were co-treated with cholesterol (20 μg/mL) and cholesterol sulfate (20 μg/mL) for 24 h. The data represent the mean ± SD (n = 3). (**C**) HaCaT cells were transfected with YPEL3 siRNA (50 nM) for 24 h and then treated with cholesterol sulfate (20 μg/mL) for 24 h. The data represent the mean ± SD (n = 3). (**D**) HaCaT cells were transfected with pcDNA3.1-STS (3 μg) for 24 h and then treated with cholesterol sulfate (20 μg/mL) for 24 h. (**E**) STS knock-down HaCaT cells were treated with cholesterol (20 μg/mL) for 24 h. The data represent the mean ± SD (n = 3). (**F**–**J**) HaCaT_STS^+/−^ cell line was constructed using the CRISPR/Cas9 system in HaCaT cells. (**F**) HaCaT_STS^+/−^ cells were seeded at 1, 2, 3, 4, or 5 × 10^4^ cells/well in a 24-well plate and incubated in medium containing 10% FBS for 72 h. Cell viability was measured using CCK assay. The data represent the mean ± SD (n = 3). (**G**) HaCaT_STS^+/−^ cells were incubated with 10% FBS + DMEM medium for 6 days. Cell viability was measured using CCK assay. The data represent the mean ± SD (n = 3). (**H**) STS and YPEL3 protein levels were measured using western blot analysis. (**I**) Confocal analysis was performed to assess YPEL3 expression. Microscopy scale bar = 50 μm. (**J**) HaCaT_STS^+/−^ cells were transfected with YPEL3 siRNA (50 nM) for 48 h. Real-time qPCR was performed to detect the expression of YPEL3 mRNA. The data represent the mean ± SD (n = 3). *p < 0.05.

To find new target genes controlled by the accumulation of cholesterol sulfate when STS deficiency occurs, we performed RNA-seq analysis using HaCaT cells treated with cholesterol (20 μg/mL) or cholesterol sulfate (20 μg/mL) for 24 h. We have identified a total of 22 genes that were increased by cholesterol sulfate and decreased by cholesterol, or vice versa (Fig. 1A and Table 1), in particular, the expression of YPEL3 changed the most. Keratinization is the process of causing the terminal differentiation of keratinocytes, and finally leading to cell cycle arrest of keratinocytes and moving to the G_0_ phase. Previous report showed that YPEL3 induces cell cycle arrest in breast cancer^19^, thus, it was hypothesized that increased expression of YPEL3 by cholesterol sulfate may be involved in keratinization. To confirm the gene controlled by cholesterol or cholesterol sulfate, real-time qPCR was performed using HaCaT cells co-treated with cholesterol and cholesterol sulfate for 24 h. It was confirmed that the expression of YPEL3 was increased by approximately 1.7-fold in cholesterol sulfate-treated cells, and this increase was prevented by cholesterol (Fig. 1B). However, unlike the results of the RNA-seq analysis, there was no change in YPEL3 mRNA levels in cells treated with cholesterol alone. To ensure that the upregulation of YPEL3 was caused by cholesterol sulfate, HaCaT cells were treated with cholesterol sulfate (20 μg/mL) for 24 h in the presence of YPEL3 siRNA (50 nM) (Fig. 1C). We found that depletion of YPEL3 mRNA by siRNA was rescued by cholesterol sulfate. To confirm that YPEL3 was suppressed when the expression of STS, which catalyzes the hydrolysis of cholesterol sulfate, was increased, HaCaT cells were transfected with pcDNA3.1_STS expression vector (3 μg) for 24 h, and then treated with cholesterol sulfate (20 μg/mL) for 24 h (Fig. 1D). The results show that YPEL3 levels increased by cholesterol sulfate were not decreased by STS overexpression. Finally, HaCaT cells in which the expression of STS was reduced by STS shRNA were treated with cholesterol (20 μg/mL) for 24 h, and then YPEL3 mRNA levels were determined (Fig. 1E). The results show that inhibition of STS induced the expression of YPEL3, whereas cholesterol did not show any significant effect. Collectively, these data demonstrate that inhibition of STS may induce YPEL3 expression through the accumulation of cholesterol sulfate in human keratinocytes.

**Table 1:** HaCat cells were treated with cholesterol (20 μg/mL) or cholesterol sulfate (20 μg/mL) for 24 h.

Gene symbol	Fold change		Chromosome	Start	End	Width	Strand	Transcript_id	Description
	Cholesterol	Cholesterol sulfate							
YPEL3	0.64	2.34	chr16	30,103,635	30,107,537	1426	−	NM_031477	Yippee like 3
DOCK8	0.61	1.79	chr9	214,865	465,259	7505	+	NM_203447	Dedicator of cytokinesis 8
ZNF428	0.53	1.76	chr19	44,111,376	44,124,014	1395	−	NM_182498	Zinc finger protein 428
CSAG3	0.66	1.76	chrX	151,876,743	151,928,738	2877	−	NM_001129825_1	CSAG family member 3
COL9A2	0.59	1.71	chr1	40,766,163	40,782,939	2831	−	NM_001852	Collagen type IX alpha 2
MIR1260B	0.66	1.67	chr11	96,074,502	96,074,690	89	+	NR_036125	MicroRNA 1260b
FOXO4	0.61	1.53	chrX	70,315,999	70,323,384	3183	+	NM_001170931	Forkhead box O4
ACOT1	0.58	1.59	chr14	74,003,928	74,010,498	1603	+	NMI_001037161	Acyl-CoA thioesterase 1
BPGM	0.64	1.56	chr7	134,331,531	134,364,567	2230	+	NM_001293085	Bisphosphoglycerate rnutase
COMMD6	0.59	1.52	chr13	76,099,350	76,123,575	3646	−	NM_203497	COMM domain containing 6
CXCL6	1.53	0.64	chr4	74,702,273	74,704,477	1659	+	NM_002993	C-X-C motif chemokine ligand 6
LYRM4	1.62	0.62	chr6	5,108,653	5,261,183	2350	−	NR_104418	LYR motif containing 4
STRC	2.75	0.59	chr15	43,891,761	43,910,998	5515	−	NM_153700	Stereocilin
P3H2-AS1	1.84	0.54	chr3	189,838,753	189,862,635	730	+	NR_126419	P3H2 antisense RNA 1
PAPPA	1.61	0.54	chr9	118,916,071	119,164,608	10,978	+	NM_002581	Pappalysin 1
SLC16A9	1.53	0.52	chr10	61,410,522	61,469,649	3987	−	NM_194298	Solute carrier family 16 member 9
ADAT3	2.11	0.50	chr19	1,905,371	1,913,446	1626	+	NM_138422	Adenosine deaminase, tRNA specific 3
MIR648	1.69	0.47	chr22	18,463,634	18,463,727	94	−	NR_030378	MicroRNA 648
SRPX	1.53	0.46	chrX	38,008,588	38,080,177	1903	−	NM_006307	Sushi repeat containing protein, X-linked
STC2	1.61	0.43	chr5	172,741,726	172,756,506	5343	−	NM_003714	Stanniocalcin 2
SCN9A	1.58	0.37	chr2	167,051,697	167,232,497	9760	−	NM_002977	Sodium voltage-gated channel alpha subunit 9
ABCA1	1.93	0.25	chr9	107,543,284	107,690,527	10,502	−	NM_805502	ATP binding cassette subfamily A member 1

RNA sequencing was performed using a HiSeq X10. Cut off ≥ 1.5-fold (*p* < 0.05).

### STS deficiency suppresses cell growth and induces cellular senescence

**Figure 2:**
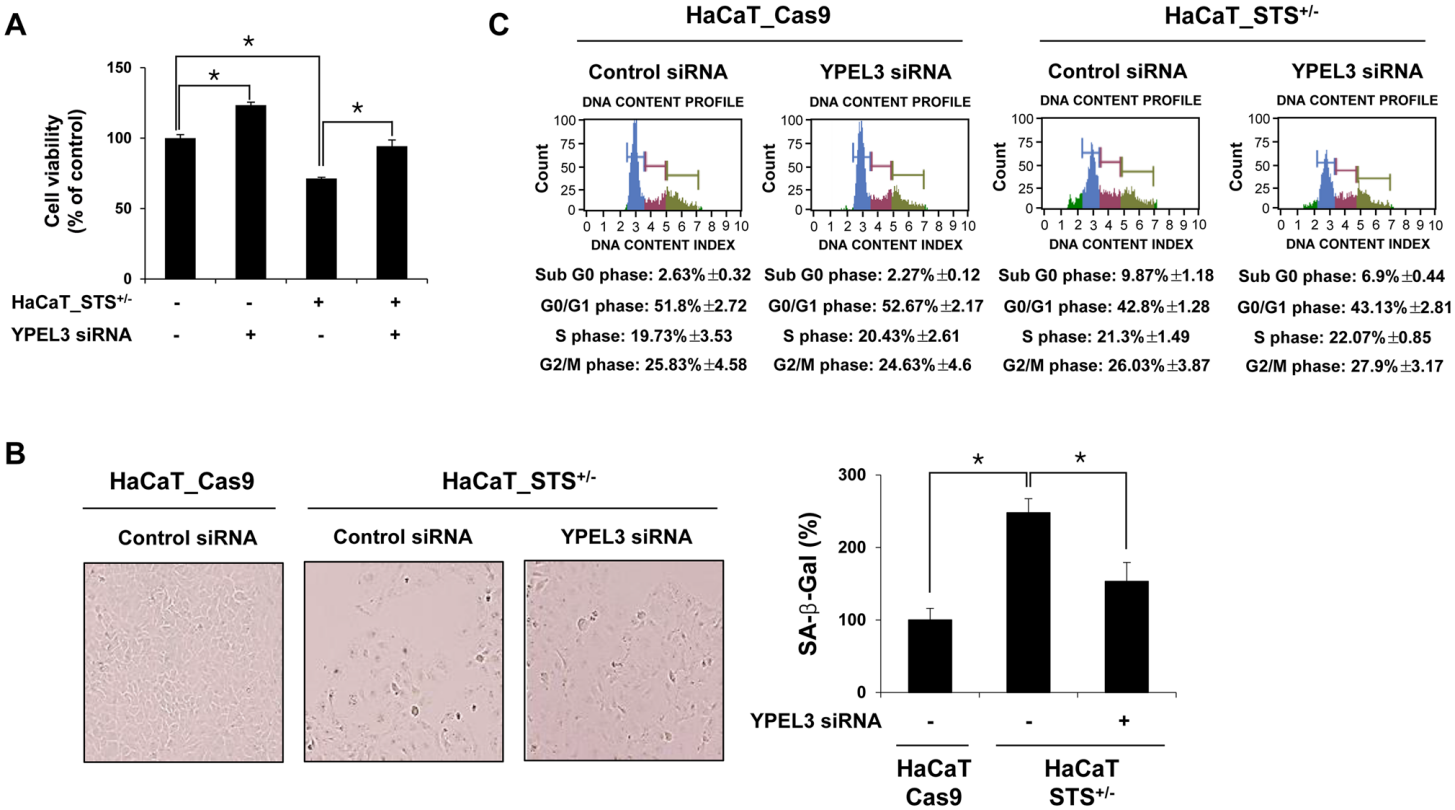
Increased YPEL3 levels by STS deficiency cause cellular senescence. (**A**) HaCaT_Cas9 and HaCaT_STS^+/−^ cells were transfected with YPEL3 siRNA (50 nM) for 48 h. Cell viability was measured using CCK assay. The data represent the mean ± SD (n = 3). (**B**) HaCaT_STS^+/−^ cells were transfected with YPEL3 siRNA (50 nM) for 48 h. Cellular senescence was measured using CST-senescence β-galactosidase staining kit. The data represent the mean ± SD (n = 3). (**C**) HaCaT_Cas9 and HaCaT_STS^+/−^ cells were transfected with YPEL3 siRNA (50 nM) for 48 h. Cell cycle was measured using Muse cell cycle kit. The data represent the mean ± SD (n = 3). *p < 0.05.

To determine whether a persistent STS deficiency affects the expression of YPEL3 in human keratinocytes, we attempted to construct STS knock-out (STS^−/−^) cells using the CRISPR/Cas9 system and targeting the exon 5 region. In several trials, we found that cells with full STS knock-out (STS^−/−^) showed a significant cell death, and only STS^+/−^ cells could be isolated as a single cell clone. In HaCaT_STS^+/−^ cells, suppression of cell growth was also confirmed when cell density was low (Fig. 1F). When HaCaT_STS^+/−^ cells were incubated for 6 d without medium change, cell growth was significantly inhibited after 4 d (Fig. 1G). To confirm that YPEL3 protein levels were increased in HaCaT_STS^+/−^ cells, western blotting and confocal microscopy analysis were performed (Fig. 1H and 1I). YPEL3 protein levels were upregulated in HaCaT_STS^+/−^ cells in both analyses. Interestingly, YPEL3 siRNA considerably inhibited the increased YPEL3 mRNA levels in HaCaT_STS^+/−^ cells (Fig. 1J). Previous studies have shown that YPEL3 induces cellular senescence in U2OS osteosarcoma and MCF-7 breast cancer cells^19^. To determine whether YPEL3 induced by STS deficiency affected cell growth and senescence in human keratinocytes, cell viability, senescence, and cell cycle were determined using YPEL3 siRNA-transfected HaCaT_STS^+/−^ cells. In HaCaT_STS^+/−^ cells, cell viability was increased by YPEL3 siRNA (Fig. 2A). Similar to what is shown Fig. 2A, the induction of cellular senescence was decreased by YPEL3 siRNA (Fig. 2B). The cell cycle assay results show that sub G_0_ phase cells were increased from 2.63% to 9.87% when comparing HaCaT_Cas9 (control cells) and HaCaT_STS^+/−^ cells. In addition, when HaCaT_STS^+/−^ cells were transfected with YPEL3 siRNA (50 nM) for 48 h, sub G_0_ phase cells were decreased from 9.87% to 6.9% (Fig. 2C). Taken together, our data suggest that upregulation of YPEL3 by STS deficiency causes inhibition of cell growth and induction of senescence in human keratinocytes.

### STS Deficiency induces YPEL3 expression through ERα inhibition

**Figure 3:**
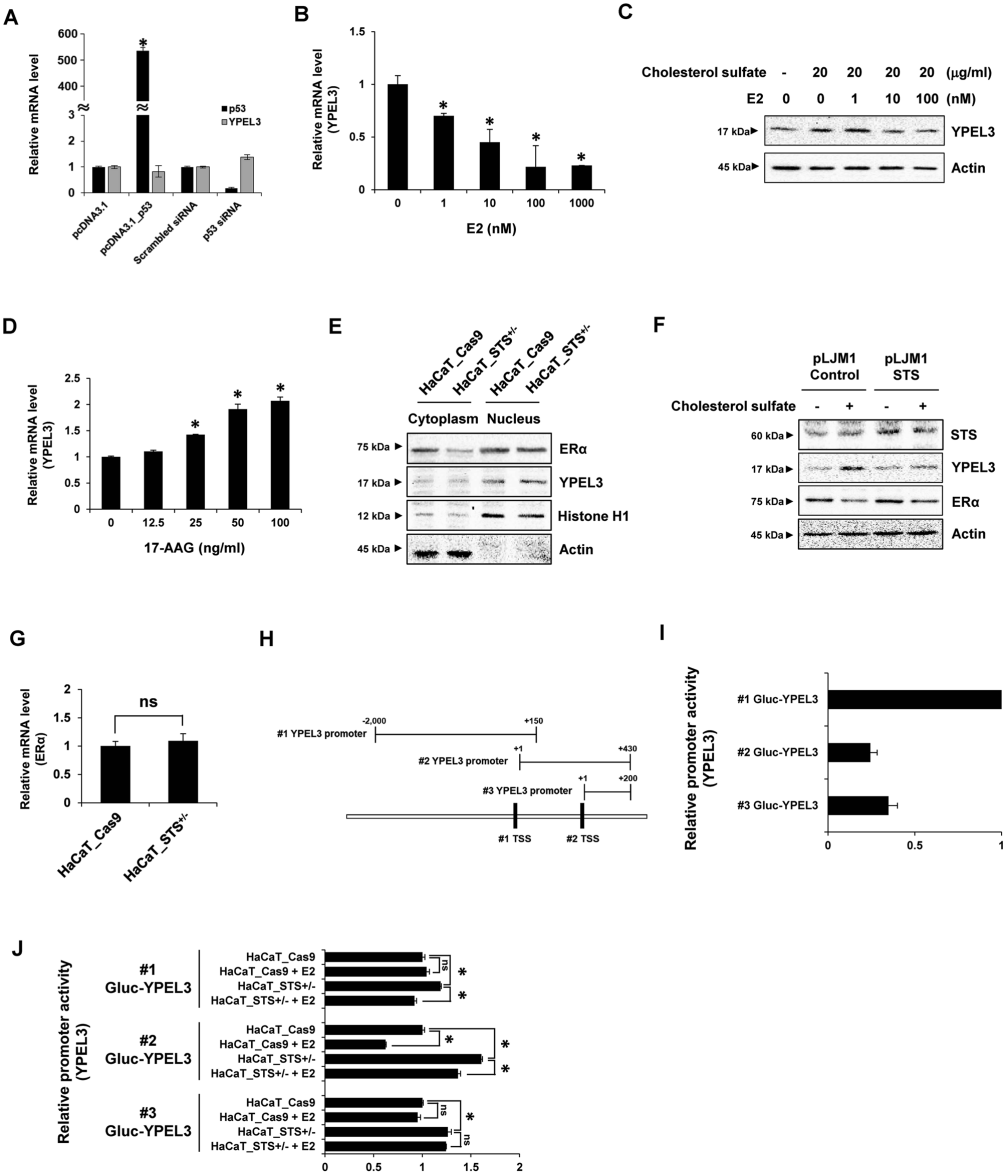
Reduced ERα levels by STS deficiency induce YPEL3 expression in a p53-independent manner. (**A**) HaCaT cells were transfected with pcDNA3.1-p53 (3 μg) or p53 siRNA (30 nM) for 48 h. Real-time qPCR was performed to detect the expression of p53 and YPEL3 mRNA. The data represent the mean ± SD (n = 3). (**B**) HaCaT cells were treated with E2 (1, 10, 100, or 1,000 nM) for 24 h. Real-time qPCR was performed to detect the expression of YPEL3 mRNA. The data represent the mean ± SD (n = 3). (**C**) HaCaT cells were co-treated with E2 (1, 10, or 100 nM) and cholesterol sulfate (20 μg/mL) for 24 h, YPEL3 protein level was measured using western blot analysis. (**D**) HaCaT cells were treated with 17-AAG (12.5, 25, 50, or 100 ng/mL) for 48 h. Real-time qPCR was performed to detect the expression of YPEL3 mRNA. The data represent the mean ± SD (n = 3). (**E**) Nuclear and cytosolic lysates were subjected to western blot analysis for ERα and YPEL3. (**F**) pLJM1_STS cells were treated with cholesterol sulfate (20 μg/mL) for 24 h, ERα and YPEL3 protein levels were measured using western blot analysis. (**G**) Real-time qPCR was performed to detect the expression of ERα mRNA. The data represent the mean ± SD (n = 3). (**H**) Schematic diagram for candidates of YPEL3 promoter sequence. (**I**) HaCaT cells were transfected with Gluc-YPEL3 reporter vectors (#1, #2, or #3; 5 μg) for 48 h. Promoter activities were measured using Gaussia luciferase (Gluc) and secreted alkaline phosphatase (SEAP) dual-reporter system. (**J**) HaCaT_Cas9 and HaCaT_STS^+/−^ cells were transfected with Gluc-YPEL3 promoter vectors (5 μg) for 24 h and then treated with E2 (100 nM) for 24 h. Promoter activity was measured using Gluc and SEAP dual-reporter system. The data represent the mean ± SD (n = 3). *Ns* not significant; *p < 0.05.

A previous study has shown that p53 plays a crucial role in inducing expression of YPEL3 in MCF-7 and U2OS cells^16^. To confirm that changes in p53 expression regulate the expression of YPEL3 in human keratinocytes, YPEL3 mRNA levels were determined after transfection with p53 expression vector (3 μg) or p53 siRNA (30 nM) for 48 h in HaCaT cells (Fig. 3A). However, contrary to expectations, expression of YPEL3 was not regulated by p53 in HaCaT cells. Because Tuttle et al. showed that estrogen repressed expression of YPEL3 in ER-positive breast cancer, HaCaT cells were treated with β-estradiol (E2; 1, 10, 100, or 1000 nM) for 24 h to determine whether YPEL3 mRNA levels were regulated by E2 (Fig. 3B)^19^. The results show that mRNA levels were downregulated by E2 in a concentration-dependent manner. To see that YPEL3 induced by cholesterol sulfate was suppressed by E2, cells were co-treated with cholesterol sulfate (20 μg/mL) and E2 (1, 10, or 100 nM) for 24 h (Fig. 3C). The results showed that YPEL3 levels induced by cholesterol sulfate are decreased by E2. Moreover, to evaluate the effects of ER, it was determined whether YPEL3 expression was upregulated by 17-(allylamino)-17-demethoxygeldanamycin (17-AAG; 12.5, 25, 50, or 100 ng/ml), an inhibitor of heat shock protein 90 (HSP90), for 48 h (Fig. 3D). 17-AAG is known to bind to the ATP-binding pocket of HSP90 and convert its function to promote the degradation of steroid receptors^24,25^. As expected, 17-AAG significantly induced the expression of YPEL3 in HaCaT cells. To confirm that ERα protein levels were regulated by STS deficiency, we compared cytosolic and nuclear levels of YPEL3 in HaCaT_Cas9 and HaCaT_STS^+/−^ cells (Fig. 3E). The results showed that ERα protein levels are downregulated by STS deficiency in the cytosolic fraction. However, there was no significant change in ERα levels in the nuclear fraction. To confirm whether accumulation of cholesterol sulfate decreases the expression of ERα, and whether STS overexpression prevents the ERα reduction by cholesterol sulfate, STS-overexpressing (pLJM1_STS) cells were treated with cholesterol sulfate (20 μg/mL) for 24 h (Fig. 3F). In the control cells treated with cholesterol sulfate, the expression of ERα was suppressed, whereas YPEL3 expression was increased. However, ERα suppression by cholesterol sulfate was decreased in STS-overexpressing cells. Unlike the changes in ER protein levels resulting from STS deficiency, no change in mRNA levels were observed (Fig. 3G). These data suggest that the accumulation of cholesterol sulfate by STS deficiency reduces ERα protein levels and increases YPEL3 levels in keratinocytes.

### Transcription of YPEL3 is increased through a P53-independent pathway by STS deficiency

The results in Fig. 3A–G showed that the increase in YPEL3 expression by STS deficiency occurs through a decrease in ERα expression. To determine whether STS deficiency increased the transcription of YPEL3 and where the transcription factors are bound to the promoter, three reporter vectors containing the two sequences expected to be the transcription start site (TSS) of YPEL3 were prepared (Fig. 3H). After measuring the promoter activity of the three reporter vectors, the #1 Gluc-YPEL3 reporter (− 2000 to + 150) showed the largest luciferase activity (Fig. 3I). Next, the HaCaT_STS^+/−^ cells were treated with E2 (100 nM) to determine the changes in transcriptional activity for each promoter sequence. Unlike the largest signal intensity in #1 Gluc-YPEL3 reporter, the relative changes in transcriptional activity of YPEL3 by STS deficiency or E2 were greatest in #2 Gluc-YPEL3 reporter (Fig. 3J). These results suggest that YPEL3 transcription is initiated from the #1 putative TSS, and the binding site of the unidentified transcription factor modulated by ERα is located between #1 and #2 putative TSSs. In addition, according to Kelley et al., the p53 binding site in the YPEL3 promoter is located upstream of #1 putative TSS^16^. Therefore, these data show that regulation of YPEL3 expression through the STS-ERα signaling pathway is independent of p53.

### YPEL3 upregulates keratinization markers INV and LOR

**Figure 4:**
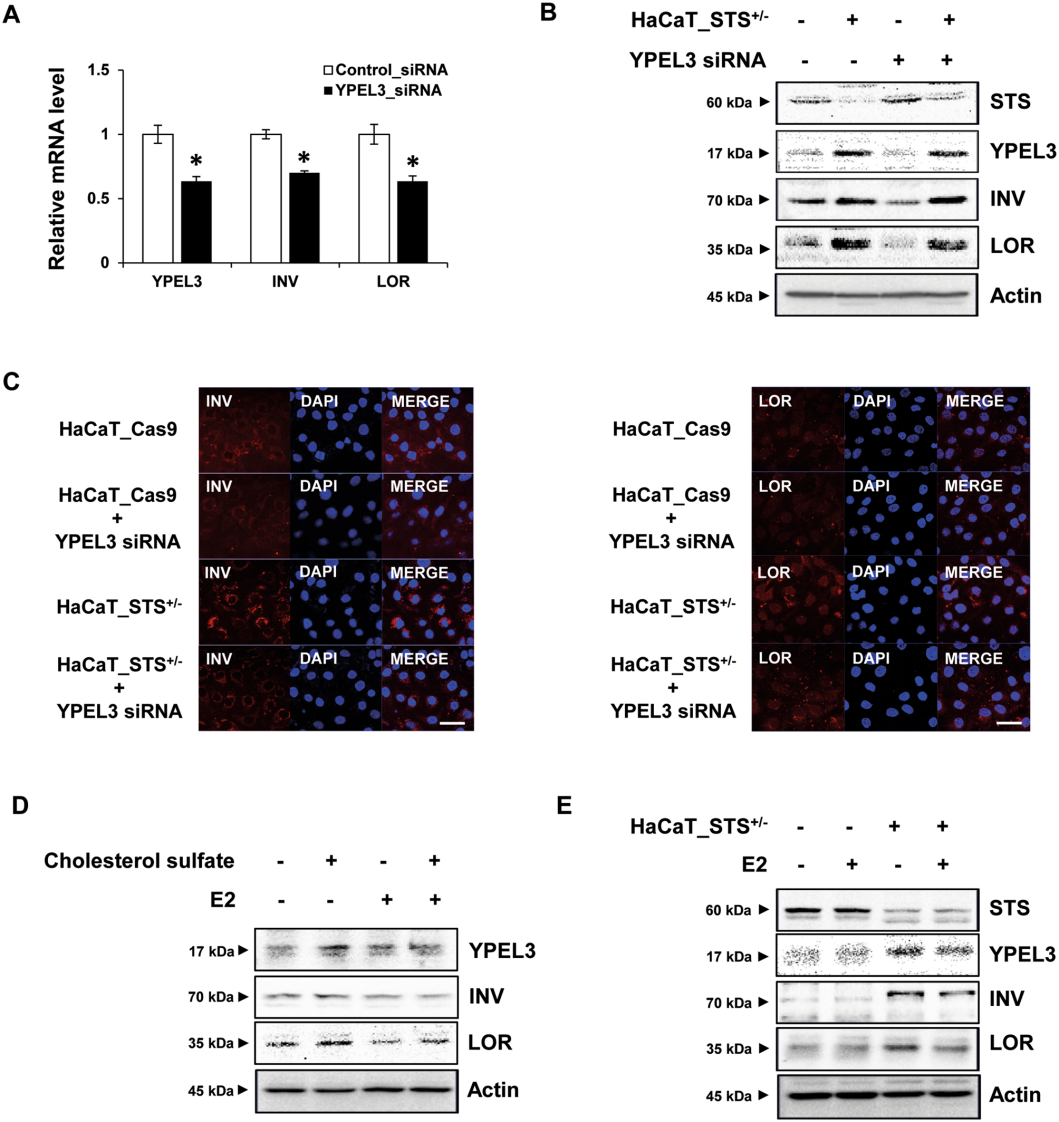
High YPEL3 levels in HaCaT_STS^+/−^ cells increase the protein levels of keratinization markers LOR and INV. HaCaT_STS^+/−^ cells were transfected with YPEL3 siRNA (50 nM) for 48 h. (**A**) Real-time qPCR was performed to detect the expression of YPEL3, INV and LOR mRNA. The data represent the mean ± SD (n = 3). (**B**) STS, YPEL3, INV and LOR protein levels were measured using western blot analysis. (**C**) Confocal analysis was performed to assess INV and LOR expression. Microscopy scale bar = 50 μm. (**D**) HaCaT cells were co-treated with cholesterol sulfate (20 μg/ml) and E2 (100 nM) for 24 h. YPEL3, INV and LOR protein levels were measured using western blot analysis. (**E**) HaCaT_STS^+/−^ cells were treated with E2 (100 nM) for 24 h. STS, YPEL3, INV and LOR protein levels were measured using western blot analysis. *p < 0.05.

**Figure 5:**
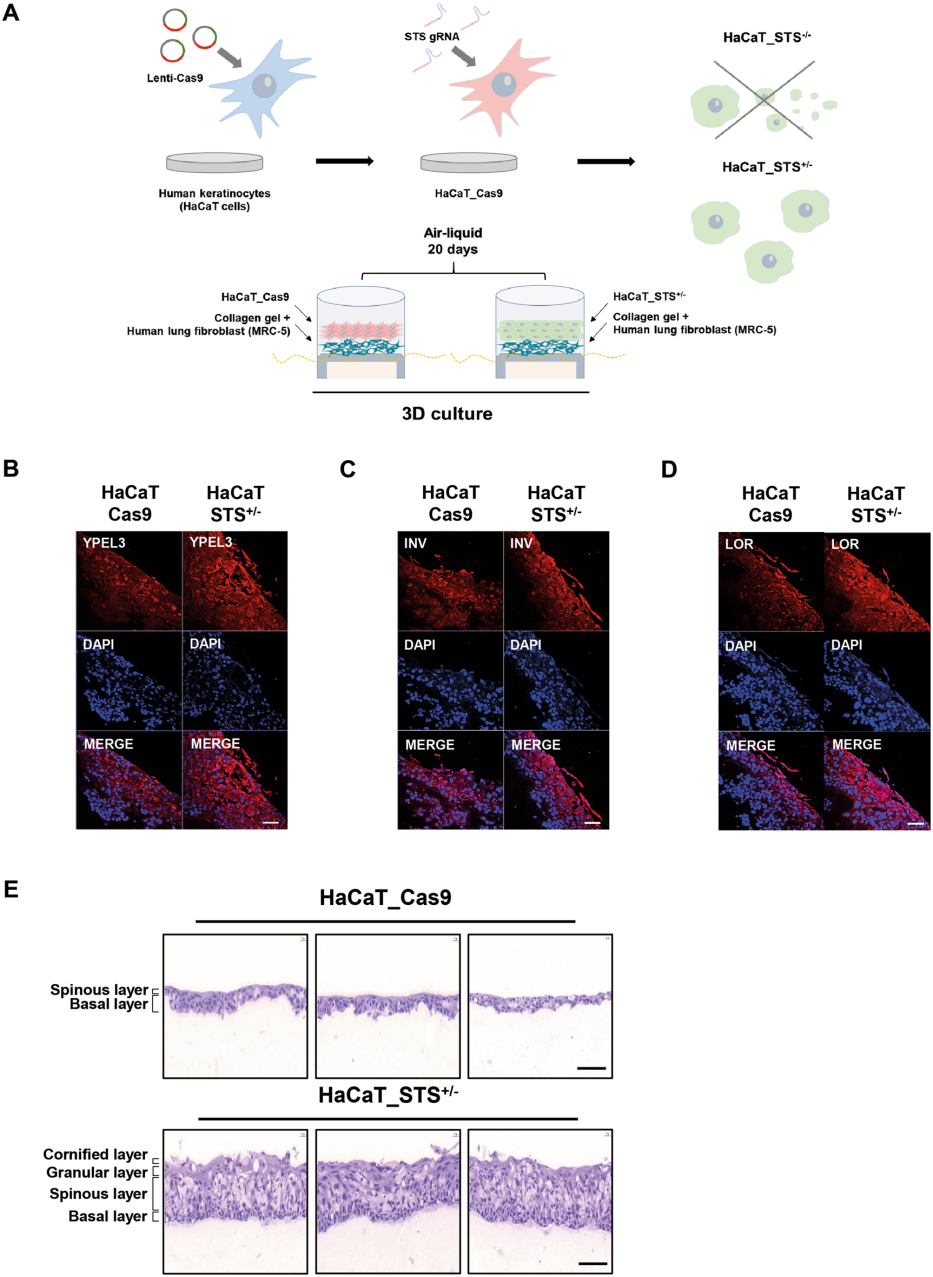
3D skin culture using HaCaT_STS^+/−^ cells. (**A**) Schematic diagram of 3D culture using HaCaT_STS^+/−^ cells. Confocal analysis was performed to assess (**B**) YPEL3, (**C**) Involucrin, (**D**) Loricrin expression. Microscopy scale bar = 50 μm. (**E**) Differences of formation of skin-layer were measured by H&E staining. Microscopy scale bar = 100 μm.

Through the results described above, we found that STS deficiency increased the expression of YPEL3. In previous studies, it was confirmed that cellular senescence causes abnormal cell differentiation. Therefore, to determine whether YPEL3 plays an important role in regulating the expression of keratinization markers such as INV and LOR, HaCaT cells were transfected with YPEL3 siRNA for 48 h and then INV and LOR mRNA levels were determined (Fig. 4A). The results showed that the expression of INV and LOR decreases in the same pattern as that of YPEL3. To determine the corresponding protein levels, HaCaT_Cas9 and HaCaT_STS^+/−^ cells were transfected with YPEL3 siRNA for 48 h (Fig. 4B). YPEL3 siRNA significantly reduced INV and LOR levels in HaCaT_Cas9 cells. However, the effect was quite small in HaCaT_STS^+/−^ cells. Confocal microscopic analysis also showed similar results (Fig. 4C). To confirm that intracellular E2 affects the decrease in INV and LOR expression, HaCaT cells were co-treated with E2 (100 nM) and cholesterol sulfate (20 μg/mL) for 24 h (Fig. 4D). The results show that E2 significantly reduced INV and LOR protein levels, whereas these inhibitory effects were reduced in cells co-treated with cholesterol sulfate. In addition, the upregulation of INV and LOR in HaCaT_STS^+/−^ cells was also suppressed by E2 (Fig. 4E). These data suggest that ERα-mediated signaling may play an important role in suppressing the expression of INV and LOR through YPEL3 reduction in keratinocytes. To identify the changes in differentiation factors in an environment similar to that occurring in a patient with an XLI disease, 3D culture was performed using human lung fibroblast MRC-5 cells and keratinocyte HaCaT_Cas9 and HaCaT_STS^+/−^ cells (Fig. 5A). We then determined YPEL3, INV, and LOR levels using confocal microscopic analysis, and the difference in composition of skin layers between normal and STS deficiency keratinocytes using H&E staining. The expression of YPEL3, INV, and LOR were increased in STS-deficient 3D culture cells (Fig. 5B–D). Compared to normal keratinocytes, granular and spinous layers approximately fivefold thick were observed in 3D culture using HaCaT_STS^+/−^ cells (Fig. 5E), although the excess cornified layer, a symptom of XLI, was not found. These data suggest that STS deficiency plays a crucial role in abnormal differentiation of keratinocytes through ERα-YPEL3 signaling. We believe that 3D cell culture using STS-deficient cells is the first XLI disease research model described, and it will enable effective validation of therapeutic candidates for XLI.

## Discussion

Previous studies have shown that mutation in the STS gene or accumulation of intracellular cholesterol sulfate causes a refractory skin disease called XLI^26–29^. However, the detailed role of STS in XLI and the cellular signaling pathways involved have not been fully elucidated. In this study, we have performed RNA-seq analysis using HaCaT cells treated with cholesterol sulfate to find new factors expected to contribute to XLI disease. We found a total of 22 genes, 10 of which were upregulated and 12 downregulated when cells were treated with cholesterol sulfate. YPEL3, which is known to promote cellular senescence, was found among the 22 candidate genes. In experiments to determine whether abnormal expression of STS affected YPEL3 expression, we found the YPEL3 levels were increased in STS-deficient cells. However, because the effects of STS overexpression and cholesterol itself were not significant, regulation of YPEL3 expression is proposed to be mainly a result of STS deficiency.

Estrogen reduced the expression of YPEL3 independently of p53, and we found that E2 reduced YPEL3 mRNA and protein levels. In several previous studies, it was identified that HSP90 inhibitors, such as geldanamycin (GA) and 17-AAG, bind to the ATP-binding region of HSP90 and change its function^30–32^. Altered HSP90 promotes degradation of ER and progesterone receptor (PR) through reducing protein stability^33^. We confirmed that YPEL3 expression was increased by 17-AAG. Moreover, ERα protein levels in cytosolic fraction were significantly downregulated in STS-deficient cells, whereas ERα mRNA levels remained mostly unchanged. Accordingly, we hypothesized that the increased expression of YPEL3 by STS deficiency is regulated by ER signaling, and the decrease in ERα protein levels may occur through regulation of factors affecting ERα protein stability such as mucin 1 (MUC1), protein interacting with never in mitosis A (PIN1), glycogen synthase kinase-3 (GSK3), and ring finger protein 31 (RNF31)^34–39^. In addition, although we found that E2 reduced promoter activation of YPEL3 and identified which sites of YPEL3 promoter were targeted, it is still unclear which transcription factor binds to this site. One possibility is that when ERα expression in the cytoplasm decreases, signal transducer and activator of transcription 5A (STAT5a) is translocated to the nucleus, resulting in YPEL3 transcription. In the previous study, Dai et al. showed that STAT5a/PARPγ pathway induces keratinocytes differentiation and regulates the expression of involucrin, a terminal differentiation marker in keratinocytes^40^. Also, it has confirmed that ERα binds to STAT5a in cytoplasm and inhibits the phosphorylation of STAT5a and translocation to the nucleus^41^. These results may suggest that the decrease in ERα expression due to the deficiency of STS increases the nuclear translocation of STAT5a, leading to differentiation of keratinocytes. Therefore, further study is needed to determine the association between STAT5a and YPEL3 expression, and the relationship between factors affecting ERα stability and STS.

Jang et al. showed that senescent oral keratinocytes have upregulated differentiation markers such as INV and transglutaminase^42^. Our results show that INV and LOR levels were upregulated in STS-deficient cells, and the upregulation of INV and LOR was suppressed by transfection with YPEL3 siRNA or treatment with E2. Therefore, it seems that the deficiency of STS increases the expression of INV and LOR through upregulating YPEL3 levels, and the ER signaling pathway plays a crucial role in this regulation. However, the mechanism whereby YPEL3 increases the expression of INV and LOR remains unclear.

Currently, XLI studies have been conducted mainly using samples from patients^43–45^. Thus, only limited analyses such as metabolite levels in the serum or gene expression patterns are possible. In a previous study, mice with STS gene deficiency were reported, but were not intentionally generated by targeting specific sequences in the X chromosome. Furthermore, research models using human keratinocytes are not available to date. Several researchers have used 3D skin models similar to patient’s skin to study certain diseases^46–49^. In particular, 3D skin models targeting psoriasis were mainly used, whereas 3D skin models targeting XLI are not available yet. In this study, we induced a partial deletion of the STS gene in keratinocytes using the CRISPR/Cas9 system and used it to construct a 3D skin model similar to the skin of XLI patients. As a result, in the 3D skin model using STS-deficient cells, an increase in the spinous layer was noticeable. The transformation from basal layer to spinous layer is an early stage of keratinization, and an excessive increase in the spinous layer ultimately leads to an abnormal formation of the cornified layer.

To summarize, we have shown that the accumulation of cholesterol sulfate by STS deficiency induces YPEL3 expression, which in turn promotes cellular senescence and abnormal differentiation. Second, that reduction of ERα levels in STS-deficient cells is a major cause of increased expression of YPEL3. Accordingly, YPEL3 may be used as a target for the development of XLI-specific therapies. Finally, the STS-deficient 3D skin model obtained using the CRISPR/Cas9 system may be an effective strategy for evaluating the efficacy of substances targeting XLI disease.

## Materials and methods

### Reagents

Cholesterol and E2 were purchased from Sigma-Aldrich (St. Louis, MO), cholesterol sulfate from Avanti Polar Lipids (Birmingham, AL), and 17-AAG from Enzo Life Science (Farmingdale, NY). All chemicals except cholesterol (using ethanol) were prepared in dimethyl sulfoxide, stored as small aliquots at − 20 °C, and then diluted as needed in cell culture media. DMEM media were purchased from HyClone (Logan, UT). FBS and charcoal-stripped FBS were purchased from Tissue Culture Biologicals (Long Beach, CA), Neon transfection system from ThermoFisher Scientific (Waltham, MA), and D-Plus ECL solution from Dongin LS (Seoul, Korea). Anti-STS polyclonal antibody (ab62219), anti-Involucrin polyclonal antibody (ab53112), and anti-Loricrin polyclonal antibody (ab85679) were purchased from Abcam (Cambridge, MA). β-Actin (sc-4778), goat anti-rabbit IgG-Texas Red (sc-2780), and Ultra Cruz mounting medium (sc-24941) were purchased from Santa Cruz Biotechnology (Santa Cruz, CA). Anti-ERα polyclonal antibody (A0296) was purchased from Abclonal (Woburn, MA) and anti-YPEL3 polyclonal antibody (15403-1-AP) from Proteintech (Chicago, IL). All other chemicals were obtained from commercial sources.

### Cell culture

HaCaT human keratinocyte cell line was obtained from CLS Cell Lines Service (Germany). MRC-5 human lung fibroblast cell line was obtained from the Korean Cell Line Bank (KCLB, Korea). HaCaT cell line was cultured in DMEM supplemented with 10% (v/v) FBS, 100 U/mL penicillin, and 100 μg/mL streptomycin. HaCaT_Cas9 and HaCaT_STS^+/−^ cell lines were cultured in DMEM supplemented with 20% (v/v) FBS, 100 U/mL penicillin, and 100 μg/mL streptomycin. Cells were incubated at 37 °C in a humidified atmosphere of 5% CO_2_. For treatment with cholesterol, cholesterol sulfate, or E2, cells were seeded in growth medium. After 24 h, the medium was changed to DMEM with 10% (v/v) charcoal-stripped FBS, 100 U/mL penicillin, and 100 μg/mL streptomycin. Cells were incubated for 72 h and then treated with cholesterol, cholesterol sulfate, or estradiol for 24 h.

### Transient and stable transfection

YPEL3 siRNA (Bioneer, Korea) and the pcDNA3.1/Zeo + vector containing the STS coding sequence were used for transient transfection. Cells were transfected with 30 nM siRNA or 3 μg plasmid with the Neon Transfection System (Life Technologies, CA). The pLJM1-Empty (a gift from Joshua Mendell, Addgene plasmid # 91980), pLKO.1 puro (a gift from Bob Weinberg, Addgene plasmid #8453), pMD2.G, and psPAX2 (a gift from Didier Trono, Addgene plasmid # 12259 and #12260) plasmid vectors were obtained from Addgene^20,21^. HEK293T cells were co-transfected with the pLJM1-STS, pMD2.G, and psPAX2 vectors. After 48 h, the medium containing the lentiviral STS gene was collected, and HaCaT cells were subsequently treated with lentiviral supernatant containing STS gene and polybrene (8 μg/mL) for 24 h. HaCaT cells overexpressing STS were selected using puromycin (1 μg/mL).

### Generation of HaCaT_STS^+/−^ cells

pLentiCas9-T2A-GFP (a gift from Roderic Guigo & Rory Johnson, Addgene plasmid # 78548) and tet-pLKO-sgRNA-puro (a gift from Nathanael Gray, Addgene plasmid # 104321) plasmid vectors were obtained from Addgene^22,23^. Lentiviral supernatant containing Cas9 gene or STS sgRNA were prepared using HEK293T cells. HaCaT_STS^+/−^ single cell was separated using 96 well plates.

### Quantitative PCR

qPCR was performed using the Rotor-Gene Q machine (Qiagen, Netherlands) and analyzed using Rotor-Gene Q Software 2.2.3 (Qiagen). Each reaction contained 10 μL of Q Green 2 × qPCR Master Mix, 1 μM oligonucleotide primers, and 20 ng of cDNA in a final volume of 20 μL. Amplification was conducted as follows: one cycle at 95 °C for 5 min, followed by 40 cycles of denaturation at 95 °C for 15 s and annealing/extension at 56 °C for 45 s. The following primer sets were used for qPCR: YPEL3, 5′-GTGCGGATTTCAAAGCCCAAG-3′ and 5′-CCCACGTTCACCACTGAGTT-3′; 18S rRNA, 5′-GTAACCCGTTGAACCCCATT-3′ and 5′-CCATCCAATCGGTAGTAGCG-3′; Involucrin, 5′-TCCTCCAGTCAATACCCATCA-3′ and 5′-CAGCAGTCATGTGCTTTTCCT -3′; Loricrin, 5′-TCATGATGCTACCCGAGGTTTG-3′ and 5′-CAGAACTAGATGCAGCCG-GAGA-3′; and ERα, 5′-GGGAAGTATGGCTATGGAATCTG-3′ and 5’-TGGCTGGACACATAT-AGTCGTT-3′.

### Gluc and SEAP dual-luciferase reporter assay

Gluc-YPEL3 reporter vectors were constructed by inserting YPEL3 promoter constructs. YPEL3 promoter constructs contained nt − 2000 to + 150 or + 1 to + 430 from #1 TSS or nt + 1 to + 200 from #2 TSS, respectively. Gluc-YPEL3 reporter vectors included Gaussia luciferase (Gluc) and SEAP genes downstream of the YPEL3 and CMV promoters, respectively. HaCaT cells were transfected with 5 μg Gluc-YPEL3 reporter vectors for 48 h and then media were harvested for measuring Gluc and SEAP activity. Gluc and SEAP activities were measured using Secrete-Pair Luminescence assay kit (GeneCopoeia). Luminescence intensities of Gluc and SEAP were measured at 480 nm using a FilterMax F3 multi-mode microplate reader (Molecular Devices, CA).

### Western blotting analysis

Cells were solubilized with ice-cold RIPA buffer containing 50 mM NaF. Extracts were separated by SDS-PAGE on 8 or 12% polyacrylamide gels and then electrophoretically transferred to 0.45 µm PVDF membranes. Membranes were blocked with 5% (w/v) BSA in Tris-buffered saline containing 0.1% Tween-20 (TBST) for 2 h at 4 °C and then incubated with primary antibodies at a 1:1000 dilution in TBST. After incubation with secondary antibodies for 2 h, proteins were visualized using the D-Plus ECL solution (Dongin LS, Korea) and analyzed using ChemiDoc XRS (Bio-Rad, CA).

### Cell viability assay

Cells (2 × 103 cells/well) were seeded in 96-well plates and incubated for 1–6 d or transfected (5 × 10^3^ cells/well) with YPEL3 siRNA (50 nM) were seeded in 96-well plates and incubated for 72 h. EZ-CyTox solution (DoGenBio, Korea) was added to each plate and then incubated for 2 h. Cell viability was measured using spectrophotometry at 450 nm and a Sunrise™ microplate reader (Tecan, Männedorf, Switzerland).

### Cell cycle assay

Cells were harvested with 0.1% trypsin–EDTA, washed twice with PBS, and re-suspended with 70% cold ethanol. After fixation, cells were washed twice with PBS and cells were stained with Muse Cell Cycle Assay kit. Cell cycles were measured with a Muse cell analyzer (Merck Millipore, Germany).

### Senescence β-galactosidase assay

HaCaT_STS^+/−^ cells were transfected with YPEL3 siRNA (50 nM) and incubated in 6-well plates for 72 h. Cells were stained overnight at 37 °C using a senescence β-galactosidase staining kit (Cell Signaling Technology, MA).

### 3D skin culture with dermal equivalent

For dermis construction, collagen gel matrix and MRC-5 cell mixture were incubated in 12 mm Millicell cell culture inserts (Merck Millipore) for 5 day. HaCaT_Cas9 and HaCaT_STS^+/−^ cells were cultured in DMEM/F12 (1:1) medium containing hydrocortisone (0.4 μg/mL), gentamycin (100 μg/mL), insulin (5 μg/mL), and ascorbic acid (50 μg/mL). HaCaT cells were cultured for 5 day, followed by additional incubation for 20 day under an air–liquid interface.

### Immunofluorescence

Samples were fixed with 10% neutral formalin for 30 min at 24 °C and blocked for 45 min in PBS containing 10% goat serum and 0.2% Triton X-100, and then incubated with primary antibody (1:200) overnight at 4 °C and stained overnight with goat anti-rabbit IgG-Texas Red (1:200). A PBS wash was performed three times at all stages. Coverslips were mounted on glass slides using Ultra Cruz mounting medium. Fluorescence was analyzed using an LSM 800 confocal laser scanning microscope (Carl Zeiss, Germany).

### Hematoxylin and eosin staining

3D culture samples were fixed in 10% neutral formalin. Samples were cut into 3-μm thick sections, sequentially dehydrated in xylene, 99.9%, 95%, 80%, and 70% ethanol, and subsequently stained with hematoxylin and eosin. All steps were performed using an automatic H&E stain Tissue-Tek, Prisma E2 (SAKURA, Japan).

### RNA-seq

Libraries were prepared from total RNA using the NEBNext Ultra II Directional RNA-Seq Kit (New England Biolabs, UK). mRNA isolation was performed using the Poly(A) RNA Selection Kit (Lexogen, Austria). Isolated mRNAs were used for cDNA synthesis and shearing following the manufacturer’s instructions. Indexing was performed using Illumina indexes 1–12. The enrichment step was carried out using PCR. Subsequently, libraries were checked using the Agilent 2100 bioanalyzer (DNA High Sensitivity Kit) to evaluate the mean fragment size. Quantification was performed using the library quantification kit and a StepOne Real-Time PCR System (Life Technologies, CA). High-throughput sequencing was performed as paired-end 100 sequencing using a HiSeq X10 (Illumina, CA).

### Statistical analysis

Dunnett’s pairwise multiple comparison t-test was performed using GraphPad Prism 4 software (GraphPad Software, CA). Differences were considered statistically significant at *p < 0.05.

## Supplementary Information

Supplementary Information.

## Abbreviations

STS: Steroid sulfatase
DHEAS: Dehydroepiandrosterone sulfate
E1S: Estrone sulfate
XLI: X-linked ichthyosis
RNA-seq: RNA sequencing
YPEL3: Yippee-like 3
INV: Involucrin
LOR: Loricrin
17-AAG: 17-(Allylamino)-17-demethoxygeldanamycin
HSP90: Heat shock protein 90
TSS: Transcription start site
GA: Geldanamycin
MUC1: Mucin 1
PIN1: Protein interacting with never in mitosis A
GSK3: Glycogen synthase kinase-3
RNF31: Ring finger protein 31
STAT5a: Signal transducer and activator of transcription 5A
