# Supplementary material for: Steroid sulfatase deficiency causes cellular senescence and abnormal differentiation by inducing Yippee-like 3 expression in human keratinocytes
Source: Sci Rep. 2021 Oct 21;11:20867. doi: 10.1038/s41598-021-00051-w (PMC8531280; doi:10.1038/s41598-021-00051-w)
Supplement: Supplementary file 1 — Supplementary Information. [file 41598_2021_51_MOESM1_ESM.pdf]

Steroid sulfatase deficiency causes cellular senescence and abnormal differentiation by inducing Yippee-like 3 expression in human keratinocytes

Hyoung-Seok Baek[1ǂ], Tae-Uk Kwon[1ǂ], Sangyun Shin[1], Yeo-Jung Kwon[1] & Young-Jin Chun[1*]

[1]College of Pharmacy and Center for Metareceptome Research, Chung-Ang University, Seoul, Republic of Korea 06974

ǂ Both authors contributed equally to this work.

*Corresponding author. Prof. Young-Jin Chun, College of Pharmacy and Center for Metareceptome Research, Chung-Ang University, Seoul, Republic of Korea 06974

E-mail address: yjchun@cau.ac.kr (Y.J. Chun)

Original panel for each western blot

Figure 1. H

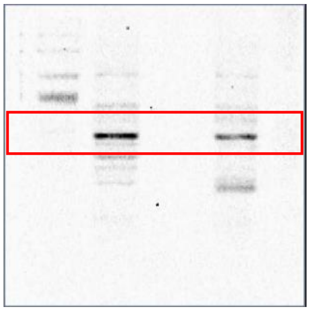

STS

Figure 1. H

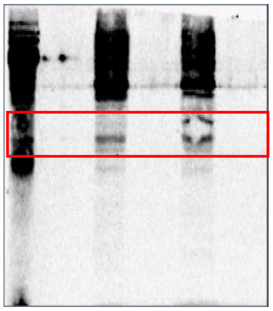

YPEL3

Figure 1. H

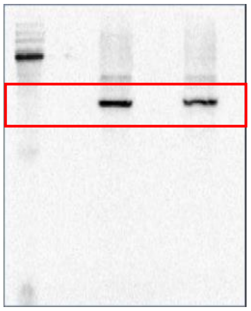

Actin

Figure 3. C

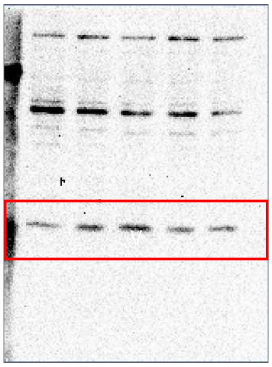

YPEL3

Figure 3. C

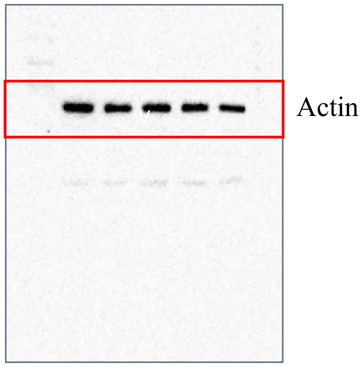

Actin

Figure 3. E

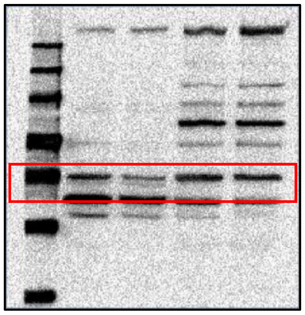

ERα

Figure 3. E

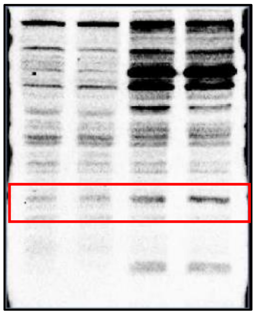

YPEL3

Figure 3. E

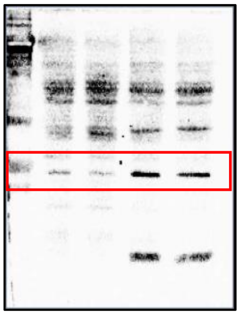

Histone H1

Figure 3. E

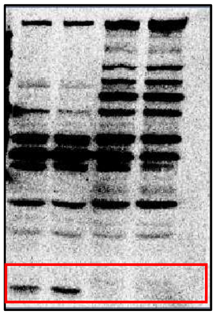

Figure 3. F

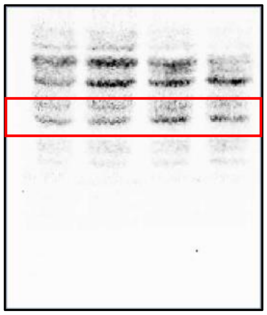

Figure 3. F

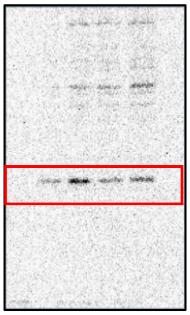

Figure 3. F

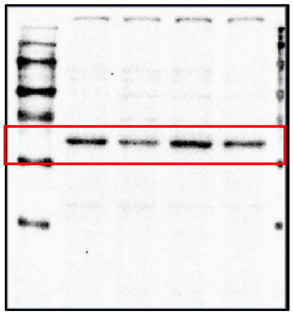

Figure 3. F

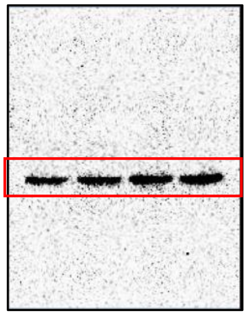

Figure 4. B

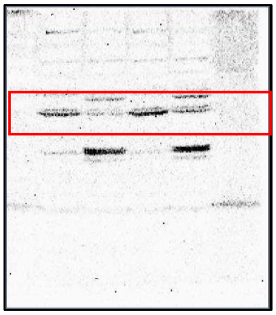

STS

Figure 4. B

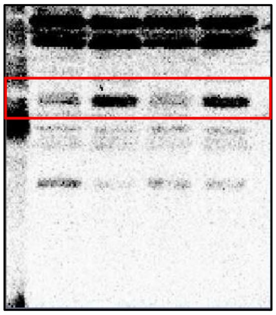

YPEL3

Figure 4. B

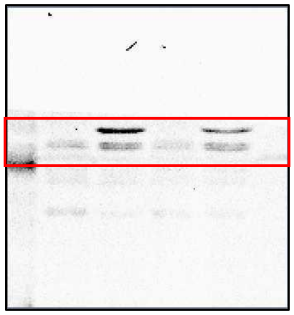

LOR

Figure 4. B

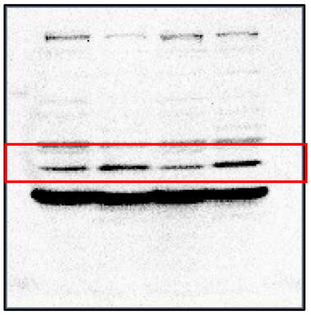

INV

Figure 4. B

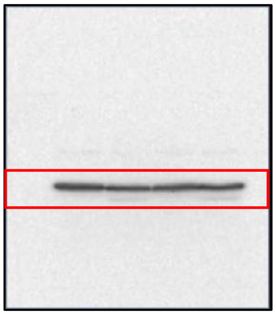

Actin

Figure 4. D

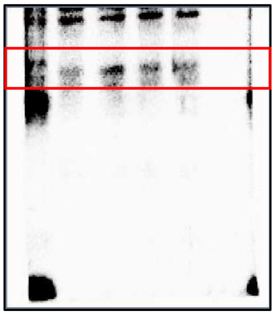

YPEL3

Figure 4. D

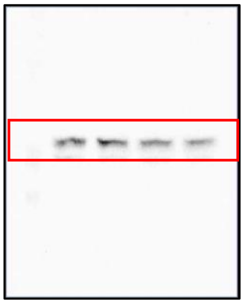

INV

Figure 4. D

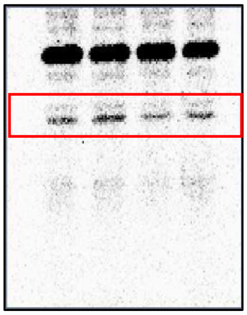

LOR

Figure 4. D

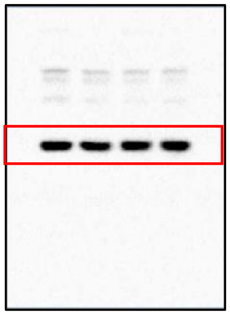

Actin

Figure 4. E

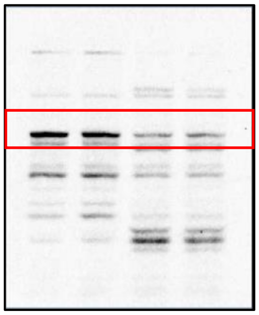

STS

Figure 4. E

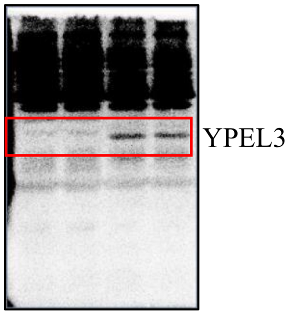

YPEL3

Figure 4. E

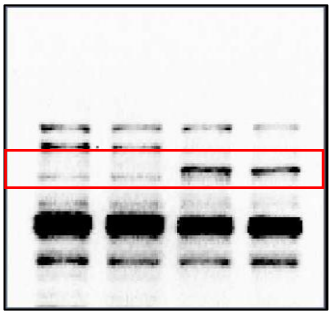

INV

Figure 4. E

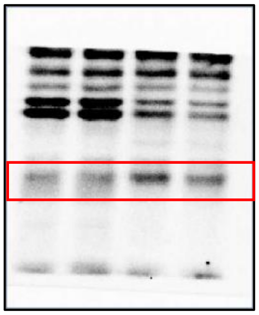

LOR

Figure 4. E

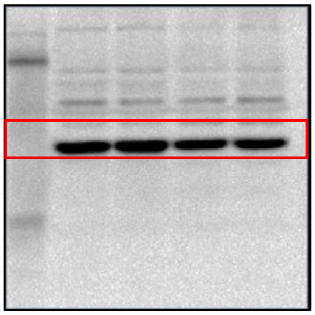

Actin
